# Habituation mechanisms and their importance for cognitive function

**DOI:** 10.3389/fnint.2014.00097

**Published:** 2015-01-08

**Authors:** Susanne Schmid, Donald A. Wilson, Catharine H. Rankin

**Affiliations:** ^1^Anatomy and Cell Biology, University of Western OntarioLondon, ON, Canada; ^2^Emotional Brain Institute, Nathan Kline Institute for Psychiatric ResearchOrangeburg, NY, USA; ^3^Department of Physiology and Neuroscience, New York University Medical CenterNew York, NY, USA; ^4^Department of Child and Adolescent Psychiatry, New York University Medical CenterNew York, NY, USA; ^5^Department of Psychology, University of British ColumbiaVancouver, BC, Canada; ^6^Brain Research Centre, University of British ColumbiaVancouver, BC, Canada

**Keywords:** habituation, sensorimotor gating, sensory filtering, synaptic mechanisms, behavioral plasticity, learning and memory

How does our brain form and store memories? One way to approach this mystery is to study a very basic form of learning—habituation—in a relatively simple nervous system. Habituation describes the progressive decrease of the amplitude or frequency of a motor response to repeated sensory stimulation that is not caused by sensory receptor adaptation or motor fatigue. A multitude of different organisms, behaviors, and experimental approaches have been used to study habituation, but still surprisingly little is known about the underlying mechanisms. A theoretical framework of the concept of habituation has been laid by Thompson and Spencer (1966), and by the dual process theory of Groves and Thompson (1970), which describes habituation and sensitization as two independent processes that interact to yield the final behavioral outcome. In a symposium in 2009, the original concept was revisited and the definitions of habituation (and dishabituation) were slightly revised for clarity; however, remarkably there were only few changes to the defining characteristics (Rankin et al., 2009). It is becoming evident that behavioral habituation is caused by different mechanisms depending on time frame of stimulation, type of sensory pathway studied, and hierarchical level of signal processing. On the other hand, habituation mechanisms seem to be highly conserved, underlining the importance of habituation for the survival of a species (see Schmid et al., 2010). The scope of this Frontiers Research Topic is to give an overview over the concept of habituation, the different animal and behavioral models used for studying habituation mechanisms, as well as the different synaptic and molecular processes suggested to play a role in behavioral habituation.

Fischer et al. (2014) studied short-term habituation of the gill-withdrawal reflex in Aplysia. In accordance with the notion of different mechanisms mediating habituation in different time frames and different pathways, they report an intrinsic mechanism that is specific for short-term habituation at short training intervals of 1s. Typlt et al. (2013b) investigated the role of a voltage-and calcium activated potassium channel (BK channel) in short- and long-term habituation of an elicited behavior (acoustic startle) versus a motivated exploratory behavior using transgenic mice, and further confirm disparate, yet evolutionary highly conserved habituation mechanisms. Pilz et al. (2014) tackled a contentious issue of whether long-term habituation of acoustic startle in mice is context specific. They report that long-term habituation is stimulus-modality specific, but not context specific, confirming it as a non-associative form of learning. Dutta and Gutfreund (2014) review data from barn owls and primates on computation of saliency in the optic tectum/superior colliculus and how this is linked to habituation and neural adaptation. Perez-Gonzalez and Malmierca (2014) review different forms of spike adaptation in auditory neurons of different levels of auditory processing hierarchy. These mechanisms lead to sensory filtering and habituation of perception. Manella et al. (2013) studied how the modulatory norepinephrine system in the brain influences odor habituation and odor memory in rats.

Besides the importance of understanding the underlying mechanisms of habituation as a basic form of learning or sensory filtering, some articles go beyond understanding mechanisms of habituation and explore how its disruption impacts other cognitive domains and higher cognitive function. Typlt et al. (2013a) link habituation deficits to impairments in spatial learning. The Mini Review of De Luca (2014) sheds light on the mechanism of the habituation phenomenon of mesolimbic and mesocortical dopamine transmission in response to taste stimuli, and its putative role as a marker of cortical dysfunction in specific conditions such as addiction. Related to this topic, Lloyd et al. (2014) review the habituation of reinforcer effectiveness and the role of dopamine neurotransmission in habituation to the reinforcer. They indicate that behavioral disorders such as obesity or attention deficit hyperactivity disorder (ADHD) may be caused by abnormal habituation to the reinforcer due to genetic or environmental factors.

Interestingly, studying the electrodermal orienting reflex in humans, Steiner and Barry (2014) argue against the dual-process theory's explanation that dishabituation is caused by sensitization, and instead suggest that dishabituation is a disruption of the habituation process, with its magnitude determined by the corresponding arousal level. It is certainly debatable to what extent this can be generalized to other modalities and pathways. In a theoretical essay Cevik (2014) argues that the impact of a stimulus on behavior and its potential to modulate the effects of other stimuli increase as its distance from the body decreases, an interesting and certainly also debatable concept.

In summary, this research topic contains original research articles, reviews, and theoretical essays that provide an updated view on different models for studying habituation, its underlying mechanisms, and its importance as prerequisite for higher cognitive function. The number and high quality of the papers on this topic provide support for the notion that habituation is a rich area of study, touching on a number of important questions related to behavioral plasticity.

## Conflict of interest statement

The authors declare that the research was conducted in the absence of any commercial or financial relationships that could be construed as a potential conflict of interest.
